# The development of CD8 T-cell exhaustion heterogeneity and the therapeutic potentials in cancer

**DOI:** 10.3389/fimmu.2023.1166128

**Published:** 2023-05-18

**Authors:** Junfeng Zhang, Feifei Lei, Huabing Tan

**Affiliations:** ^1^Department of Basic Research, Guangzhou Laboratory, Guangzhou, China; ^2^Lab of Liver Disease, Department of Infectious Diseases, Renmin Hospital, Hubei University of Medicine, Shiyan, Hubei, China

**Keywords:** CD8^+^ T-cell exhaustion, heterogeneity, antitumor immunotherapy, developmental trajectory, immunity

## Abstract

CD8^+^ T cells are essential lymphocytes with cytotoxic properties for antitumor immunotherapy. However, during chronic infection or tumorigenesis, these cells often become dysfunctional with a gradually depleted ability to release cytokines and the exhibition of reduced cytotoxicity, the state referred to as “T-cell exhaustion” (Tex). This unique state was characterized by the increasing expression of inhibitory checkpoint receptors, and interventions targeting immune checkpoint blockades (ICBs) have been considered as a promising strategy to stimulate T-cell killing. Recent investigations have demonstrated that exhausted T cells not only display functional, metabolic, transcriptional, and epigenetic differences but also comprise a heterogeneous group of cells. In this review, we summarize the current findings on dynamic differentiation process during Tex heterogeneity development in cancer and chronic infection. We discuss how the responses to immunotherapy are determined by these distinct subsets and highlight prospective approaches for improving the efficacy of ICB therapy for cancer by leveraging the heterogeneity of T cells.

## 1 Introduction

The coexistence of T cells with growing tumors from human patients, which was the so-called “Hellstrom paradox” by Hellstrom et al. in 1968 (1), indicates that cytotoxic T cells become dysfunctional during tumorigenesis. It has been well documented that T-cell exhaustion (Tex), which was initially described as the hyporesponsiveness of CD8^+^ T cells during chronic infection (2, 3), can also occur in persistent antigen stimulation and in a variety of cancers (4–6). Reducing cytokine release and cytotoxicity, a sustained expression of inhibitory receptors (IRs), and hyporesponse to tumor and chronic infection are pointed out as the hallmark of Tex (7–9). It is now accomplished that the immune system utilizes Tex to adapt to the persistent antigen stimulation, therefore limiting the loss function of the immune system during infection or disease. The therapeutic blocking of IR pathways to reinvigorate Tex cells has been considered to be one promising strategy to treat certain types of cancer (10). However, most patients still show limited efficacy to immune checkpoint blockades (ICBs) in clinical practice. A subsequent study emerged that exhausted T cells were not terminally differentiated state derived from late-stage effector cells but are rather composed of a remarkable heterogeneous population with subsets retaining self-renewal capacity and responsiveness to ICBs. Moreover, recent studies by utilizing high-dimensional single-cell technology on antigen-specific Tex populations (11, 12) in humans and mice (11–15), have highlighted a more complex heterogeneity of exhausted CD8^+^ T cells, including a major subset of Tex progenitor cells (Tpex) that are responsive for checkpoint blockade (7, 14, 16, 17). Emerged evidence suggests that Tex heterogeneity is maintained in a dynamic balanced state during chronic infection and tumorigenesis. Thus, the defined different subsets that are involved in Tex heterogeneity were raised from a continuous development process, and the development of heterogeneity during CD8 Tex is highly relevant to the Tex heterogeneous state. Thus, a better understanding of this process could provide us insight into the connections from self-renewing progenitors to effector-like or terminally exhausted cells. However, the developmental trajectory and mechanisms governing their fate decisions remain incompletely understood. Fully understanding the development hierarchy of Tex has significant implications for the efficacy of adoptive T-cell transfer and checkpoint blockade treatments for cancer.

In this review, we summarize the recent findings on the dynamic differentiation process during Tex heterogeneity development in cancer and chronic infection. We provide a summary of the traits and molecular underpinnings of heterogeneity in exhausted CD8^+^ T cells. We discuss how the responses to immunotherapy are determined by these distinct subsets, and we highlight prospective approaches for improving the efficacy of ICB therapy for cancer by leveraging the heterogeneity of T cells.

## 2 The development of T-cell exhaustion heterogeneity

### 2.1 The phenotypical and function heterogeneity in T-cell exhaustion

In the context of cancer and chronic infection, the T-cell differentiation program is considerably changed and distinct from that of effector and memory T cells. During acute infection, the activation of naive T cells by antigen and T-cell receptor (TCR) signals happens, leading to the rapid expansion of the effector T cells. Most activated T cells die after the peak of effector expansion, with a group of effector precursors transitioning into the memory T cell (18). However, during persistent infection and tumorigenesis, the Tex represent a distinct T-cell lineage (9, 19, 20). Studies in the model of chronic LCMV infection have demonstrated that Tex cells exhibit a prolonged production of IRs under ongoing exposure to persistent antigen while drastically reducing effector activities (21–23). When compared to memory and fully functional effector T cells, the Tex cells also have altered transcriptional, epigenetic, metabolic, and differentiation programs (24). Additionally, there is a steady decline in the production of vital cytokines like TNF and IL-2 at the early stages and an insufficiency in IFN production at the late stages, despite the continued production of chemokines like MIP-1 and RANTES (24–27). Other functional defects of Tex cells also include an impaired response to hemostatic cytokines (17). Memory T cells maintain self-renewal *via* IL-7 and IL-15, independent of antigen stimulation (24). In comparison, tumor antigen–specific Tex cells, with are deficient in IL-2R and IL-7R signaling, cannot respond to IL-7 and IL-15 signaling–mediated self-renewal but instead rely on persistent antigen stimulation (28). Moreover, terminal exhausted T cells eventually lose their response to proliferation signals, as well as to antigen stimulation and the development of hemostatic memory. Those findings suggest that multiple signals from both intracellular and extracellular sources can be integrated to negatively impact T-cell differentiation, promoting Tex. Precisely balancing those signals would possibly maintain proper T-cell function under tumor microenvironments.

### 2.2 Identification of progenitors in T-cell exhaustion heterogeneity

It is widely acknowledged that Tex during tumorigenesis or chronic infections represent a heterogeneous population. Models of Tex cell emergence from early studies proposed that Tex cells represent the progeny of formerly terminally differentiated effector T cells. Recent studies have shown that exhausted T cells are a heterogeneous population; they exhibit different levels of PD-1 expression and has a unique response to PD1 blockade. The PD-1-high Tex cells do not respond to PD1 inhibition and have low effector function, in contrast to the PD-1-low Tex cells that were thought to be progenitors that can respond selectively to checkpoint blockade (29, 30). The subsequent single-cell profiling of Tex cells from cancer and chronic infection models (12–15, 31) showed distinct populations with memory-like, effector-like, and exhausted features, indicating a more complex cellular landscape. Heterogeneity has since been acknowledged as a hallmark of Tex populations (29, 32–35). More recently, Bengsch et al. developed high-resolution mass spectrometry (CyTOF) profiling to investigate the heterogeneity of Tex cells in HIV-positive or prostate-cancer patients. Nine different Tex cell clusters were identified, each displaying unique coexpression patterns for inhibitory receptors and functional, transcriptional, and phenotypic characteristics (36). These results indicate that Tex populations are much more complex than previously thought, and a comprehension of the hierarchy of Tex development might benefit in revealing the molecular and cellular mechanisms that driving T cell exhaustion.

### 2.3 The linear development model of T-cell exhaustion heterogeneity

**Figure 1:**
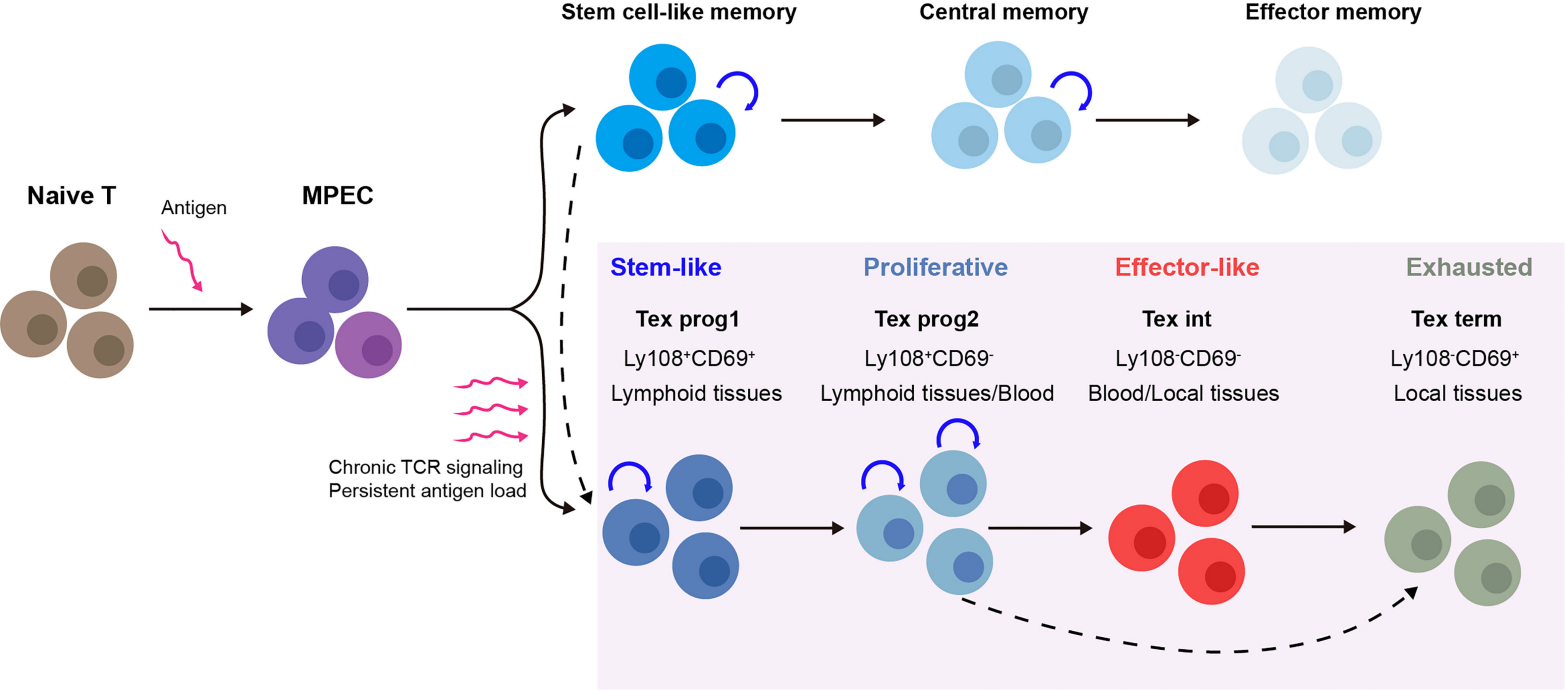
Multistep development process of T-cell exhaustion (Tex) heterogeneity. Upon acute infection or vaccination, naive T cells are activated and after the peak of effector expansion, a subset transitioning into the memory T-cell pool (18). In contrast, during chronic infection and cancer, the program of T-cell differentiation is substantially altered. Under chronic T-cell receptor (TCR) signaling or persistent antigen stimulation, antigen-specific T cells follow a stepwise linear development process that is composed of multiple levels of heterogeneity. For each stage, the subsets show distinct gene expression, function, and physical location. Tex prog1 may also develop from antigen-specific stem-like memory T cells (37), while terminal dysfunction Tex may also be derived directly from Tex progenitors under TCF1 regulation (38). MPEC, memory precursor effector cell; Tex, T-cell exhaustion; TCR signaling, T-cell receptor signaling; Tex prog1, progenitor 1 Tex subset; Tex prog2, progenitor 2 Tex subset; Tex int, intermediate Tex subset; Tex term, terminally exhausted Tex subset.

Recent studies on Tex have shown that the process of Tex may be stepwise linear and is composed of multiple levels of heterogeneity (summarized in **Figure 1**). The development hierarchy of Tex has been mapped out by Beltra et al. in 2020, which highlighted the developmental links between various subsets and provided insights into the molecular mechanisms involved in the transition of T cells (39, 40). According to their findings, Tex is a multistage developmental process, and certain stages were involved during T cells transitioned from quiescent memory-like progenitors to terminally differentiated exhausted T cells. There were four distinct subsets of exhausted T cells that were identified both in chronic infection and the solid tumor model, based on the expression of Ly108 and CD69: (1) the Tex progenitor 1 subset (Tex prog1, Ly108^+^CD69^+^) is a subset of quiescent progenitors that is restricted located in secondary lymphoid organs. This subset enriched T-cell progenitor gene (Tcf7, Myb, Il7r, and Sell) expression, indicating its self-renewing potential. (2) The Tex progenitor 2 subset (Tex prog2 and Ly108^+^CD69^-^) is a subset of high-proliferation progenitors that are located both in lymphoid tissues and blood. Also, the Tex prog2 population has an active cell cycle marker (cyclin genes and mki67) and cell motility–related gene (Anxa2, Alcam, and Itgb7) expression. (3) The Tex intermediate subset (Tex int and Ly108^-^CD69^-^) expresses cytotoxic and effector genes (Grzma, Grzmb, Prf1, Klrk1, Cx3cr1, Tbx21, Zeb2, Id2, and Prdm1) and indicates that it is an intermediate effector subset with increased effector function. The Tex int subset was found in the blood and blood-accessible tissues; it also expresses a high level of T-box transcription factor Tbx21 (Tbet), while with a reduced expression of the high-mobility group box (Tox). (4) The terminal exhausted T-cell subset (Tex term and Ly108^-^CD69^+^) shows poor proliferative activity and minimal effector function but sustains high levels of inhibitory receptor (Pdcd1, Lag3, Tigit, and Cd244) and TOX expression. The Tex term subset can be present in the blood-accessible local tissues but is absent from lymphoid tissues or circulation. These data illustrated a linear differentiation model in which a population of progenitors with a quiescent memory sequentially progresses from two intermediate stages to a terminally exhausted stage (40). Thus, the Tex populations display lineage-like characteristics and go through a sequential developmental process with a progressive loss of effector function. Additionally, it suggests that there may be a “checkpoint” at which progenitor cells that resemble quiescent memory cells decide to have progeny with an exhausted phenotype.

### 2.4 The origin of T-cell exhaustion progenitors

The origin of Tpex during Tex development remains unknown. Given that Tex progenitors are the major subset that responds to the ICBs, better understanding the development hierarchy of the Tex progenitor would facilitate in uncovering the mechanisms of Tex. Tpex are generally considered by their expression of transcriptional regulator T cell factor 1 (TCF1), a low level of inhibitory receptor PD-1, and the surface markers Ly108, CD62L, and CXCR5 are termed as the “precursors of terminal Tex effector like Tex” (Tpex) with long-term proliferative, self-renewing capacity (30, 41). Although Tex had been thought to be distinct from effector T cells and memory T cells, various studies have shown that Tpex has stem-cell and memory-like characteristics (33, 37, 39, 42–44). Here, we discuss the possible origin and characteristics of Tpex and compare it with typical T-cell memory precursors including memory stem cells, central memory cells, and tumor-specific memory T cells (summarized in **Table 1**).

**Table 1:** Comparison of the features between memory T cells and T-cell exhaustion cells.

	Stem-like memory (45)	Central memory (45)	TdLN-Ttsm* (37)	Tex prog1 (39)	Tex prog2 (39)	Tex int (39)	Tex term (39)
Markers	CD62L^+^CD45RA^+^ IL-2Rb^+^ CXCR3^+^	CD62L^+^CCR7^+^ IL-7R^+^	TCF1^+^TOX^-^PD1low	Ly108^+^CD69^+^	Ly108^+^CD69^-^	Ly108^-^CD69^-^	Ly108^-^CD69^+^
Location	LN*	LN/Blood	LN	LN	LN/Blood	Blood/tissues	Tissues
Proliferative state	+	++	+	+	++	+	-
Memory properties	++	++	++	++	+	-	-
Self-renewal	++	++	++	++	+	-	-
IR expression	Low	Low	Low	Low	Low	Moderate	High
Epigenetic imprinting	-	–	–	+/-	+	+	+

* LN, lymphoid tissues; TdLN-Ttsm, tumor-draining lymph node (TdLN)–derived tumor-specific memory cells. “+” indicates positive, “-” indicates negative. IRs, inhibitory receptors.

The Sca 1^+^ IL-2Rb^+^ CXCR3^+^ CD62L^+^ quiescent stem-like memory T cells are similar to naive T cells (45–49). After the antigen has been cleared in an acute infection, CD62L^+^ CCR7^+^ central memory T cells, effector memory T cells, or tissue resident memory T cells are preserved. Upon re-exposure to the antigen, those cells rapidly develop into functional effector cells (46). Notably, a subset of CD8^+^ T cells, which are predominantly located in T-cell zones of the lymphoid organs along with naive CD8^+^ T cells, act as stem-like/memory cells in the context of persistent infection or tumors and respond to ICB (33). However, a recent study found that the tumor-draining lymph node (TdLN)–derived tumor-specific memory cell population (TdLN-Ttsm cells) plays a crucial role in response to IR blockade in a preclinical cancer model. The majority of our knowledge about Tex was based on the studies in the content of chronic infection. Previous studies about CD8 Tex in cancer models merely focused on tumor-infiltrating lymphocytes (TILs), which mainly included terminally exhausted T cells. Recently, accumulating studies paid attention to the heterogeneity of T cells in the process of tumorigenesis, and serval new Tex subsets outside the tumor have been identified. However, the developmental trajectory for the heterogeneity during CD8 Tex in tumorigenesis is still unclear, and whether those subpopulations in the tumor model correspond to the subpopulations found in chronic infection also need further discussion (37). Remarkably, the lymph node–resident Ttsm cells showed canonical memory traits like enhanced proliferative activity and long-term persistence, and they were still able to produce effector cells with full functionality. The authors found that TCF1^+^TOX-PD1^low^ TdLN-Ttsm cells express canonical T stemness/memory-related markers, including IL7Ra, IL2Rb, and CD62L. Additionally, TdLN-Ttsm cells demonstrated strong antitumor activity upon adoptive transfer and were the main responders to checkpoint blocking. Tsui et al. intriguingly discovered that spleen-derived CD62L^+^ Tpex cells had memory stem-like features, which are essential to sustain the long-term antiviral activity and response to immunotherapy (42). In line with this finding, tumor-specific TCF1^+^PD1^low^ CD8 T cells with stem cell/memory-like features were identified in peripheral lymphoid tissues, such as spleen and lymphoid nodes recently (50–54). Moreover, Beltra et al. in 2020 revealed that the Ly108^+^CD69^+^ Tex prog1 was detected at the apex of the four-step differentiation paradigm during Tex, primarily in the peripheral lymphoid tissues with stem-cell/memory marker expression (39). Recently several studies provide evidence that the Tex progenitor may share a similar cell experience with long-lived memory T cells, i.e., exposure to low-strength TCR signaling stimulation and soluble factors from a specific microenvironment such as the lymph node paracortex region. Those findings raise the possibility that the Tex progenitor and stem cell–like memory cells may share a similar origin. The molecular mechanisms underlying the formation of heterogeneity within Teff, Tmem, and Tex populations in the acute and chronic LCMV infection models were recently revealed by Giles et al. This work revealed a number of new subsets, including a unique TCF1^+^ stem cell/memory-like progenitor subset in acute and chronic infection and a subset of Tex cells expressing effector genes (44). These studies suggest that the Tex progenitors have a more heterogeneous phenotype and that subsets of Tex progenitors, which are primarily found in secondary lymphoid tissues, maintain their memory stem-like characteristics during chronic infection or tumorigenesis and may be in charge of Tex population maintenance and Tex response to immunotherapy. Several recent investigations have characterized the TILs from human malignancies or lymphoid tissue and found the presence of TCF1^+^ TILs with stem cell–like, memory, and cytotoxic potential. More significantly, responses to checkpoint blockage were positively linked with the frequency of TCF1^+^ CD8 T cells in tumors (12, 31, 55).

## 3 The regulation of T-cell exhaustion heterogeneity

The molecular and cellular regulation during the exhaustion of CD8^+^ T cells has been extensively studied and discussed in the literature (17, 24). According to this, the emergence of Tex may involve the integration of signals from other lymphocyte cells, including CD4^+^ T cells and antigen-presenting cells, inhibitory cytokines, costimulatory and inhibitory receptors, and signals from microenvironments (12, 56, 57). Here, we merely focus on and discuss about the major cell-intrinsic factors, such as T-cell receptor (TCR) signaling, TCF1-mediated transcriptional control, epigenetic reprogramming, and metabolic regulation, that underlie the heterogeneity of Tex.

### 3.1 T-cell receptor signaling in T-cell exhaustion heterogeneity

Persistent antigen exposure is crucial for controlling CD8^+^ T-cell development and serves as a trigger for Tex. TCR and costimulator CD28 activate the MAPK/JNK/PI3K-AKT/IKK pathway during acute infection or vaccination. This is followed by the activation of multiple transcription factors, such as NFAT and FOS-JUN heterodimer (AP-1), which further activate the transcription of the effector genes, such as IL2 and IFNG (58–60). However, insufficient antigen stimulation during chronic infection or cancer at an early stage would cause the loss of costimulators and could not activate AP-1 and NFAT, thus interacting with negative regulators such as TOX, NR4A, EGR2, EGR3, IKZF2, and IRF4, promoting the dysfunction of T cells (61). Several factors such as Dapl1 were found regulating NFATc2 activation downstream of the TCR signal to regulate CD8(+) Tex and responses in cancer (62). It is interesting to note that, even after brief exposure to a chronic antigen, CD8^+^ T precursor cells could still create fully functioning CD8^+^ memory T cells. However, the Tex could not recover to normal memory T cells once they were undergoing exhaustion, even removing them from the antigen stimulation (63–66). The intensity of the TCR signal has also recently been emphasized as a factor that can control the phenotypic fate of T cells (67, 68). The authors proposed a model that the antigen-specific T-cell differentiation trajectory was driven by TCR signal strength based on the clonal lineage tracing of antigen-specific T cells by utilizing paired single-cell RNA and TCR sequencing. T-cell clones with high affinity to TCR prefer to adopt a terminal Tex fate, whereas there are clones with low-affinity toward killer cell lectin–like receptor G1 KLRG1+ Tex fate with effector function. It is notable that the CXCR3 expression level responds to TCR stimulation strength. Moreover, CXCR3-deficient T cells displayed differentiation divergence toward progenitors of stem-like memory cells and preferentially remained in the lymph node paracortex (T-cell zone) rather than traveling to the interfollicular regions (IFRs) (69, 70). The severity of Tex under conditions of cancer and chronic infection may therefore be correlated with the frequency and duration of chronic antigen load, which may be important initiating determinants in the development of Tex heterogeneity.

### 3.2 TCF1-centered regulation of T-cell exhaustion heterogeneity

Various studies have been conducted on the transcriptional network that uniquely functions during the early stages of Tex development. Recent technical developments in particular have made it possible for us to perform single-cell genome-wide analyses of gene expression. Tex cells expressed transcription factors including T-box factors, TCF1, as well as others crucial for functioning effector T cells and memory T cells. However, it has been discovered that those same transcription factors are linked to certain subsets and have a particular role in the transcriptional control of Tex cells. In order to investigate the regulatory network of effector versus exhausted T cells during the early stages of lymphocytic choriomeningitis virus (LCMV) clone-13 strain induced chronic infection, Chen et al. applied scRNA-seq, fate mapping on various sorted T-cell subsets from adoptive transfer in 2019 (38). One branching trajectory emerged from naive T cells and was projected to either TCF1^-^ or TCF1^+^ T cell subsets, according to the scRNA-seq data from naive T cells and antigen-specific T cells at an early point after infection. Surprisingly, the authors discovered a subset of TCF1^+^ T cells that had the capacity to develop into both KLRG1^+^ effector-like T cells with a high level of cytotoxicity and KLRG1^-^PD-1^+^ terminally exhausted T cells with a low level of effector activity. These results are in line with a prior report that TCF1^+^ progenitor T cells gave rise to TCF1- terminally exhausted T cells. The previous work demonstrated that, in the chronic or acute infection models, the depletion of TCF1 led to an increase in both terminal Tex and Teff fractions (35). In this study, they further demonstrated the crucial function of TCF1, which is primarily dependent on EOMES and c-Myb to maintain the exhausted T-cell pool. Tox-driven transcriptional and epigenetic alterations occur in the TCF1^+^ progenitor subset during the first 2 weeks of chronic infection (71–74). Thus, the TCF1 function might shift with time from being a Teff cell differentiation inhibitor to a Tex cell differentiation inhibitor (61). These findings demonstrate that the fate decision of exhausted CD8 T cells is driven by a transcriptional network centered on TCF1 (38). It is interesting to note that two recent studies (12, 75) demonstrated that proliferating T cells either develop into TCF1+ self-renewable T memory precursor cells or into TCF1^-^CX3CR1^+^ KLRG1^+^ effector T cells in response to the activation of naive T cells. The Tox-driven epigenetic program causes the T memory precursor cells to differentiate into TCF1^+^ PD-1^+^ self-renewable T memory–like exhausted progenitor cells, which, in turn, give rise to TCF1PD1^+^CX3CR1^low^ T transitory effector–like exhausted cells, which, in turn, continually differentiate into TCF1^-^PD-1^+^ CD101^+^ terminally T exhausted cells (11). Importantly, checkpoint blockade could enhance T memory precursor cell differentiation into both subsets, but only IL-21-mediated CD4^+^ T cells allow T memory precursor cells to differentiate into functional TCF1^-^CX3CR1^+^ KLRG1^+^ terminal T effector-like cells.

### 3.3 The epigenetic reprogramming in T-cell exhaustion heterogeneity

It has been demonstrated that epigenetic reprogramming is a key factor in determining the fate of T cells. Epigenetic imprinting has been demonstrated to play a crucial role in the development, maintenance, and activation of memory T cells (76–78), as well as Tex (9). The accessible chromatin areas of CD8^+^ T cells from chronic LCMV infection models were analyzed, and it was revealed that the progenitors of Tex cells differed from terminally developed Tex cells. These two subsets exhibit various genome-wide patterns in terms of chromatin accessibility (79). Furthermore, during cancer or chronic infection, the epigenetic landscapes of both progenitor and terminally exhausted T cells were distinct from those of either functional effector or memory T cells (19, 20, 80–82). Those findings indicate that epigenetic mechanisms are the driving force behind the preservation of the distinctive characteristics of Tex cell subsets. Therefore, the lack of persistent efficacy could be attributed to the failure of checkpoint blockade to alter the epigenetic profile of Tex cells. In line with this concept, in a chronic infection model using mouse CD8^+^ Tex cells, only minimal chromatin modifications in response to checkpoint blockade–induced reinvigoration were observed (19). Thus, exhaustion is a discrete T-cell lineage, and Tex cells are a stable, distinct cell type that are maintained in an epigenetically fixed state (9, 19, 76–79, 83–85).

The epigenetic profiles of exhausted T cells have revealed a number of transcriptional factors and epigenetic regulators. For instance, in chronic viral infections and tumors, thymocyte selection–associated high mobility group box protein (TOX), which is not necessary for the generation of effector and memory CD8^+^ T cells, is required for the formation and maintenance of CD8^+^ Tex (71–74, 86). However, in models of chronic infection, the genetic deletion of TOX merely accelerated terminal T-cell differentiation but did not completely delete Tex, which resulted in the short-term persistence of the virus-specific T cells. Similar to this, Scott A et al. showed that Tex cells remain in a dysfunctional state when depleted Tox in tumor-specific CD8^+^ cells (73). The subsequent study revealed that increased TOX expression promotes commitment to terminally differentiated Tex cells, primarily by converting persistent TCR signaling into a transcriptional and epigenetic developmental program specific to Tex cells. A recent study revealed that STAT5A antagonizes TOX in CD8+ Tex (87). Additionally, it has been demonstrated that DNA methylation contributes to the epigenetic reprogramming of T cells (80, 81, 88). New DNA methylation patterns were discovered in the work by Youngblood et al. during persistent LCMV infection in mouse antigen–specific CD8^+^ Tex cells at the late stage of effector cell development, and a similar pattern was discovered in TILs from a mouse prostate tumor model (88). Additionally, the conditional deletion of the *de novo* DNA methyltransferase 3 (DNMT3a) prevented the accumulation of specific DNA methylation patterns in CD8^+^ T cells from chronic infection models, which prevented the development of functional exhaustion compared to normal CD8^+^ T cells (89). Exhausted T cells are also regulated by additional epigenetic factors, such as the BET-family protein BRD4 and the histone lysine demethylase LSD1 (90, 91). Those results imply that developing small-molecule drugs targeting the epigenetic modifiers might help relieve Tex and boost the effectiveness of cancer immunotherapy.

### 3.4 The metabolic regulation in T-cell exhaustion heterogeneity

The key role that cell metabolism plays in controlling Tex is also indicated by the findings that various subsets of Tex cells may have varied metabolic properties (92). Although progenitor-exhausted T cells prefer to employ the mitochondrial processes of fatty acid oxidation (FAO) and oxidative phosphorylation (OXPHOS) for energy, terminally exhausted T cells with deficient glycolysis and OXPHOS mostly rely on glycolytic metabolism (25, 93). Additionally, accumulating data indicate that exhausted T cells may experience metabolic insufficiency, which could impede the effector function and result in a reduced response to immunotherapies (25, 92–96).

## 4 Therapeutic implication of T-cell exhaustion heterogeneity

**Figure 2:**
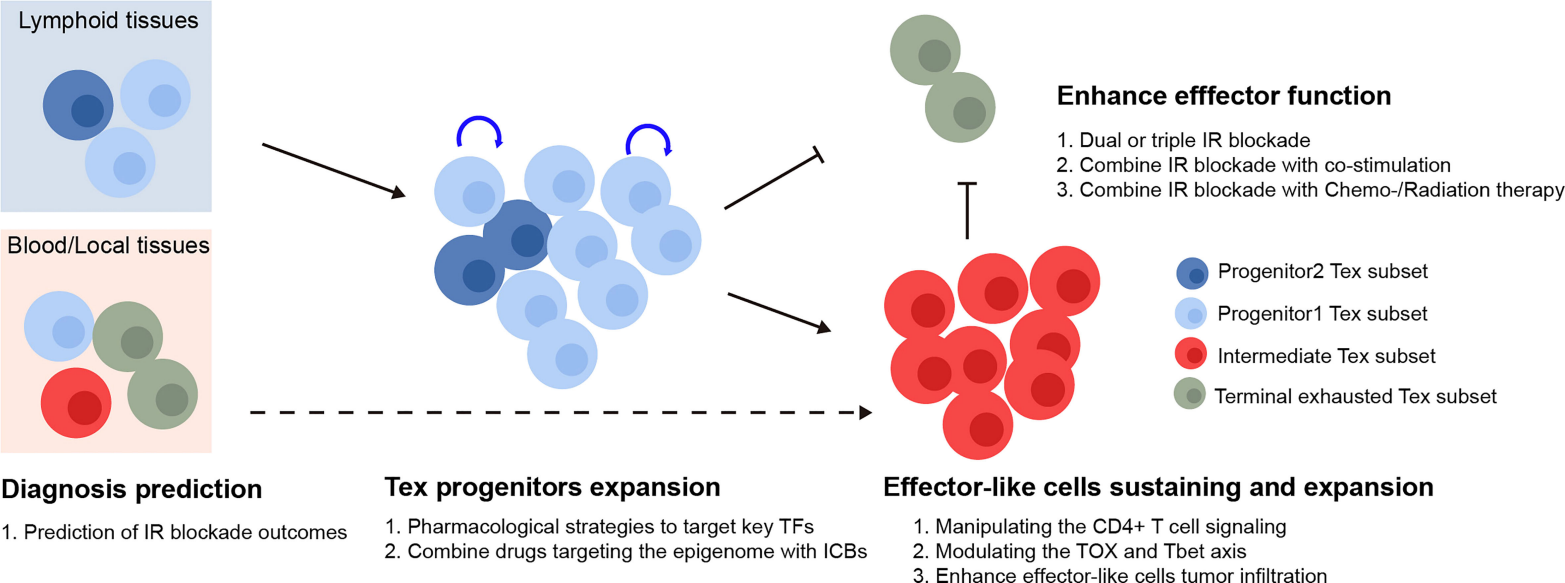
Therapeutic implication of Tex heterogeneity. The quantity of the Tex progenitor and the Tex intermediate subsets in cancer patient could potentially be used as predictors for the inhibitory receptor blockade outcomes. For therapeutic purpose, the Tex progenitors from lymphoid tissues might be expanded *via* various strategies, followed by manipulating the differentiation basis to cytotoxic Tex intermediate subsets. Finally, the Tex effector function might be further enhanced by combination therapy. TFs, transcription factors; IR blockade, inhibitory receptor blockade.

In cancer patients, immunotherapies targeting the IRs produced by Tex cells, such as PD-1, have demonstrated promising clinic potential (97). The major subsets of Tex cells have been linked to the response to checkpoint blocking, and Tex regulation has received a significant amount of focus. However, targeting the key factors to rejuvenate Tex has not been successful. Based on our current understanding of Tex heterogeneity, we describe potential ways to target various Tex subsets to increase the effectiveness of checkpoint blockade in this section (summarized in **Figure 2**).

### 4.1 Targeting the T-cell exhaustion progenitor cells

In response to checkpoint blockade, TCF1^+^PD-1^+^ tumor-specific CD8^+^ T lymphocytes showed the capability to differentiation (50). Those cells included TCF1^+^PD-1^+^ progenitors, developed cytotoxic TCF1-PD-1^+^ effector-like T cells, and terminally exhausted T cells. More importantly, the depletion of TCF1^+^PD-1^+^ TIL did limit immunotherapy responses (16), indicating that ICB relies on the propensity for the differentiation of TCF1^+^PD-1^+^ progenitor TILs rather than the ability to reverse terminally exhausted T-cell groups. According to the studies mentioned earlier, the Ly108^+^CD69^-^ Tex progenitors and Ly108^-^CD69^-^ Tex intermediate subsets were the main recipients of checkpoint blockade and had the capacity to generate cytotoxic effector cells that were similar to functioning Tex cells. Thus, the quantity of the Tex progenitor and Tex intermediate subgroups may be utilized to predict the efficacy of immunotherapies. The efficacy of checkpoint blockade may also be improved by raising the number of Tex progenitors and modifying the basis of their differentiation toward cytotoxic Tex intermediate subsets as opposed to dysfunctional terminal Tex cells, for instance, by altering the TOX and TBET axes. Various studies have shown that, after being exposed to a persistent infection during a specific time window, T cells that were exhausted could not fully recover, and the feature of exhaustion even becomes more severe with prolonged exposure (98–103). Therefore, there should be a precise treatment window to maintain the T cell, prevent it from developing a hallmark of exhaustion, and preserve its functionality. Additionally, Tex was maintained in an epigenetically fixed state even during the early stages of exhaustion development and as CD8^+^ Tex epigenetic imprinting persists even after treatment for chronic infection disease in patients (102). Hence, investigating strategies combining checkpoint inhibitors with epigenetic modification might lead to the generation of targetable Tpex with longer-term potential and more efficacy, although this hypothesis has to be further tested.

PD-1 inhibition is known to be able to transiently expand the progenitor subset of exhausted T cells (33, 34), thereby leading to the generation of more cytotoxic and terminally differentiated Tex cells. However, it also causes the loss of Tpex cells’ self-renewing ability and eventually, loss response to ICBs. Thus, exploring strategies to expand the cytotoxic subset from Tpex while maintaining its self-renewing ability would greatly improve ICB efficacy. Several transcription factors can exhibit context-dependent action, due to upstream stimulation, cofactor binding, or changed epigenetics that modify target gene locus accessibility. By manipulating CD4+ T-cell signaling to support Tex progenitors’ differentiation into functional TCF1CX3CR1+ KLRG1+ terminal effector-like T cells, it is possible to increase the bias of Texint cells differentiated into Tex effector-like cells with cytotoxic function but without terminally T exhausted cells, in accordance with the model that TCF-1 mediated the bifurcation between divergent fates of the TCF-1+Ly108+PD-1low Tex cells (12, 56).

Tumor-specific memory T (Ttsm) cells (37) or memory stem like Tpex (39) have tumor memory characteristics and can be found in peripheral lymphoid tissues, making them good potential targets for antitumor therapy. Strategies to induce, maintain, and expand these subsets could greatly enhance antitumor therapies (28, 52, 56, 104–107). Given that the TCF1+ Tpex cells are mainly located in lymphoid organs, which may provide soluble and/or direct signals to maintain their self-renewing or facility memory-like cell differentiation, modulating these signals might impact the differentiation outcomes. In line with this hypothesis, recent studies revealed that the antitumor efficacy in solid tumors could be significantly enhanced by modulating IL-9R signaling, IL-2R signaling, TGFbeta1/BMP signaling, HIF-1α signaling, and IL21 signaling in TCF1+ Tpex cells (28, 56, 105, 106, 108–111). Furthermore, a CRISPR-based screen identified the mammalian canonical BRG1/BRM-associated factor (cBAF) complex as a negative memory T-cell fate determinant, and inhibiting cBAF markedly improved efficacy in a mouse solid tumor model, suggesting that modulating the cBAF complex early during T-cell differentiation could possibly improve cancer immunotherapy (112). In summary, combining these approaches with checkpoint inhibitors may be one potential strategy for achieving improved antitumor outcomes.

### 4.2 Targeting the terminally differentiated T-cell exhaustion cells

Emerged data show that ICBs have little effect on terminally differentiated T cells (41). While CTLA-4, TIM3, and LAG3 may express at distinct phases during exhaustion, it is notable that their expression levels may vary during exhaustion. The inhibitory receptors suppress T-cell activation through a variety of mechanisms (11, 75, 113). According to those findings, the terminally differentiated Tex cells can be targeted to increase their antitumor action and appear to be necessary for the checkpoint blockade–mediated regulation of chronic infection and cancer. Combining multiple IR blockades in accordance with the time window during which they are expressed may therefore increase therapeutic effectiveness. Dual or triple IR blockades, such as PD-1/CTLA-4 and PD-1/Tim-3 blockades, may be more efficient than single IR blockades at enhancing tumor-specific CD8^+^ T-cell function. Further increasing the effector-like terminal differentiated Tex cells and enhancing antitumor results may be achieved by targeting IRs in conjunction with costimulators or in combination with chemotherapy and radiation therapy (114–117).

## 5 Future perspectives

Recent studies on the developmental trajectory of Tex, the distinctive traits of the various Tex subsets, and the ways by which they respond to checkpoint inhibitors have shed light on the immunotherapy-related mechanisms. New findings on memory stem–like Tpex or tumor-specific memory CD8^+^ T cells have greatly increased our understanding of mechanisms of response to checkpoint inhibitors. In addition to focus on TILs during tumorigenesis, tumor-specific progenitor T cells in peripheral lymphoid tissues need to be further studied to identify populations of T cells that are truly self-renewable stem cell–like/memory progenitor cells upon IR blockade. Taking advantage of the specific drivers and propagators for Tex progenitors’ fate decision, including discrete therapies together with epigenetic modifiers or upstream regulators, to boost stem-like/memory Tex progenitors and to expand effector-like T cells could provide new insights for novel therapeutic interventions to block dysfunctional Tex, hence enhancing the efficacy of immunotherapy.

## Author contributions

JZ conceived and wrote the manuscript and prepared the figures. FL contributed to performing the literature collection and manuscript revising. JZ and HT directed and approved the manuscript. All authors contributed to the article and approved the submitted version.
